# Individualized 3D-Printed Bone-Anchored Maxillary Protraction Device for Growth Modification in Skeletal Class III Malocclusion

**DOI:** 10.3390/jpm11111087

**Published:** 2021-10-26

**Authors:** Minji Kim, Jingwen Li, Sehyang Kim, Wonho Kim, Sun-Hyun Kim, Sung-Min Lee, Young Long Park, Sook Yang, Jin-Woo Kim

**Affiliations:** 1 Graduate School of Clinical Dentistry, Ewha Womans University, Seoul 07985, Korea; minjikim@ewha.ac.kr (M.K.); jingwen7883@gmail.com (J.L.); shkim0363@gmail.com (S.K.); dhsh0828@gmail.com (S.-H.K.); 2 Department of Orthodontics, School of Medicine, Ewha Womans University, Seoul 07985, Korea; kimwonho311@gmail.com; 3 Department of Oral and Maxillofacial Surgery, School of Medicine, Ewha Womans University, Seoul 07985, Korea; sm930513@dent.dku.edu (S.-M.L.); 21628@eumc.ac.kr (Y.L.P.); 4 Cusmedi Co., Ltd., Suwon-si 400815, Korea; syang@cusmedi.com

**Keywords:** bone-anchored maxillary protraction, skeletal class III malocclusion, 3D printing, growth modification

## Abstract

Bone-anchored maxillary protraction (BAMP) is effective for skeletal Class III malocclusion. However, infection, screw and plate loosening, and device failures occur with conventional plates. This pilot prospective study analyzed the feasibility of individualized BAMP using preoperative simulation and 3D titanium printing in patients referred by the orthodontic department for four BAMP miniplates. Preoperative cone beam computed tomography data were analyzed using CAD/CAM software to fabricate the individualized 3D-printed BAMP device. The customized plates were printed using selective laser sintering and inserted onto the bone through an adjunct transfer jig. The accuracy of preoperative simulation and actual placement of the BAMP device were tested by superimposing simulated positioned digital images and postoperative computed tomography data. The growth modification effect depended on superimposition of lateral cephalograms and comparative changes in SNA, SNB, ANB, and Wits. Two male patients were finally included in the study. BAMP decreased the ANB difference (−4.56 to −1.09) and Wits appraisal (−7.52 to −3.26) after 2 years. Normal measurement indices for sagittal and vertical growth indicated successful growth modification. The mean accuracy between preoperative simulation and actual surgery was 0.1081 ± 0.5074 mm. This treatment modality involving preoperative simulation and 3D titanium printing for fabricating and placing customized BAMP devices precisely at planned locations is effective for treating skeletal Class III malocclusion.

## 1. Introduction

Skeletal Class III malocclusion is a common orthodontic deformity involving a single or a combination of protrusive mandibles, deficient or retrusive maxilla, protrusive mandibular dentition, and retrusive maxillary dentition, which not only affect a patient’s masticatory function but also their esthetic appearance and mental health [1,2]. Prevalence of Skeletal Class III was reported as 7.8% [3], and Class III presents the highest prevalence among orthognathic cases [4].

In growing patients, early intervention for correcting the craniofacial relationship has been used for several decades with significant outcomes [5,6,7].

It is better to use extraoral anchorage than intraoral anchorage for skeletal growth control in the early intervention for Class III malocclusion to prevent undesirable dental changes from anchored teeth force application [8]. Among the different therapies, bone-anchored maxillary protraction (BAMP) is considered one of the most reliable methods for Class III malocclusion; it eliminates dentoalveolar effects and facilitates patient full-time wear compliance by using only intraoral skeletal anchorage. BAMP with miniplates is used in growing patients, with multiple miniscrews to ensure anchor stability and avoid any possible damage to the tooth germ [9]. A recent systematic review and clinical study showed reliable results for the treatment of skeletal Class III malocclusion, in which BAMP was reported to enhance maxillary growth and inhibit mandibular growth [10].

Despite its advantages, there are limitations to BAMP treatment, such as the need for additional surgery, possible tooth germ injury, and irritation of the adjacent tissues by elastics or miniplates. The miniplates can also loosen due to insufficient bone density at early ages. Therefore, the precise application of ready-made miniplates of sufficient bone quality following the contour of the cortical surface while avoiding the tooth germ is often surgically challenging [11,12].

Recent developments in 3D printing technology and the introduction of digital dentistry have made it possible to fabricate patient-customized simulations and individualized dental devices. In addition, metal 3D printing using selective laser sintering (SLS) allows the fabrication of individualized bone fixation plates and bone reconstruction material. BAMP is an effective treatment modality for skeletal Class III treatment; however, it is challenging to apply conventional plates onto the correct location. Therefore, the clinical feasibility of individualized BAMP plates using preoperative simulation and 3D titanium printing was tested in this pilot study.

## 2. Methods

### 2.1. Study Sample

This pilot prospective study was performed from 2019 to 2021 in departments of orthodontics and oral and maxillofacial surgery at Ewha Womans University Mokdong Hospital. This study was approved by the institutional ethics committees (EUMC 2019-06-014). Patients who were referred by the orthodontics department for four BAMP miniplates were included in the study. Any syndromic patients and cleft patients were excluded.

### 2.2. Data Acquisition

Cone beam computed tomography (CBCT) and intraoral scanning were performed to collect essential pre-surgical information to fabricate the individualized 3D-printed BAMP device. Since the BAMP device itself is placed on the maxillary and mandibular bone surface, it can be manufactured solely with CBCT, without digital information of the dentition. However, we previously experienced inaccurate placing of the BAMP device without dentitional information during the actual surgery. Therefore, an additional tooth-guided jig that can deliver the BAMP device to the precise simulated position was manufactured, and intraoral scanning was performed for an accurate tooth-guided transfer jig for properly positioning the plate.

Dicom data of CBCT were extracted into STL format and merged with intraoral scanning STL data. The CBCT dataset obtained 2 weeks before surgery was surface-rendered in the 3D model (STL format) of the bone. Intraoral Scanning with Trios3 (3 shape, Copenhagen, Denmark) started with the most distal tooth in the third quadrant and continuing to the anterior teeth. Next, the fourth quadrant was scanned, again beginning with the most distal tooth. Scanning of the maxilla started with the most distal tooth in the second quadrant and ended at the central incisor. The first quadrant was recorded starting with the most distal tooth. The camera was positioned at 45 degrees (or as close as possible to the axis of the tooth) to the buccal and lingual scans. The scanning device works by means of confocal microscopy, with a fast scanning time; the light source provides an illumination pattern to cause light oscillation on the object. As for the superimposition and merger of DICOM+STL (including software info), 3D Slicer(extension slicer RT, ver.4.11, open source) was used to create a 3D bone model file (stl format), and Meshmixer (ver.3.5, Autodesk) was used to edit the surface model created by 3D slicer.

### 2.3. Preoperative Simulation

Using Ondemand^®^ CAD/CAM software (Cybermed Co., Seoul, Korea) and Doctor Check software^®^ (Cusmedi Co., Seoul, Korea), virtual miniplates and an additional transfer jig were designed with respect to the patient’s bone contour, location of the hook, and shape of the surgical site. The maxillary plates for BAMP are preferably positioned on the zygomatico-maxillary junction and maxillary first molar. Positioning as close to the teeth as possible is more advantageous for surgical convenience and minimal surgical invasion. When located in the alveolar bone vertically too close to the teeth, there is a risk of tooth germ injury and screw loosening due to relatively more active bone remodeling and the low density of the maxillary bone during treatment. When positioned higher, near the zygomatic bone, permanent tooth germ injury can be avoided, and fixation strength can be obtained due to the greater bone density. The proper position, considering bone density for fixational support and avoiding tooth germ injury, could be determined preoperatively on the CBCT.

### 2.4. Surgical Procedure

The BAMP device consisted of a closed subperiosteal part for bone fixation and an open intraoral part for applying the elastics to deliver orthopedic force. The conventional flat-design bone fixation miniplate does not follow the bone surface contour nor does it form a tight contact with the bone, leaving small gaps that could cause biological complications. To minimize these complications, the BAMP plate is designed to be positioned to emerge intraorally at the mucogingival junction 5 mm above the gingival margin so that it can be placed along the anatomical contour when it is opened intraorally in the bone fixation part. This design minimizes the biological complications of the device maintained in the oral cavity for at least 6 months and up to 3 years. The elastics can be applied between the maxillary premolar and the first molar to maximize the patient’s treatment adherence.

Accurate application of the simulated BAMP device to the determined location during actual surgery is a challenge. An additional transfer jig can be fabricated using precise intraoral scanning data to minimize the error between simulation and actual surgery. The transfer jig is suitable for being referenced to adjacent maxillary teeth and is manufactured to carry and deliver the BAMP device. The transfer jig is manufactured using resin or titanium metal printing (SLA type, EP-3500, Shinin). The BAMP device to be placed on the mandibular anterior teeth is designed in the same manner. The mandibular device is placed on the mandibular symphysis; therefore, tooth germ injury and bone density are less of a concern than with the maxillary device.

The design of the customized BAMP device through preoperative simulation is exported as an STL file from a CAD software program (Magics, Materialise, Leuven, Belgium) and submitted for 3D printing (Metalsys150, Winforsys Co., Seoul, Korea; laser power 120–200 W) using titanium alloy (Ti6Al4V ELI, medical grade in accordance with ASTM F136, AP&C, Quebec, QC, Canada) following the SLS additive manufacturing technique. Titanium powder was melted into thin layers, which were used in the 3D printer. Post-processing of the plate involved removal of the loose power and the support structures and polishing to improve the surface quality and mechanical properties and reduce errors during CBCT and scanning data acquisition.

A rapid prototyping model of the patient was manufactured before surgery, and a virtual surgery was performed through the process of pre-fitting. In the actual surgery, customized plates were installed at the surgical site using the transfer jig as a guide. An incision (1.5–2.5 cm) was made above the bone where the fixation plate had to be placed, and minimal dissection was performed. Then, the jig was precisely matched to the reference point, indicating accurate installation of the BAMP device. After the correct position of the BAMP device was confirmed, fixation was performed using 1.5 mm screws. After confirming that the BAMP device was sufficiently fixed to the bone, the incision was sutured using vicryl 4-0 and 5-0.

Postoperatively, patients were instructed to use a chlorhexidine mouth rinse three times a day for 7 days. Analgesics and non-steroidal anti-inflammatory drugs were prescribed. After 7–10 days of soft tissue healing, Class III elastics were applied with an initial force of ~70 g on each side. The force of the elastics was increased to 150 g after 1 month of traction and again increased to 200–250 g after 3 months. Patients were instructed to wear the elastics full-time, except for meals and for brushing. They had to replace the elastics daily, with regular follow-up. Patients were also instructed to follow-up once a month postoperatively.

### 2.5. Cephalometric and Superimposition Analysis

Simulated positioned digital images and postoperative CT data were superimposed for comparison to evaluate the accuracy of preoperative simulation and actual application of the fabricated BAMP device. In addition, lateral cephalometric analysis was performed before BAMP treatment and 2 years postoperatively to evaluate the treatment effects of BAMP’s skeletal Class III correction. The superimposition of lateral cephalograms was analyzed, and changes in SNA, SNB, ANB, and Wits were compared to determine the effect of growth modification.

The 3D deviation between the position of the BAMP designed with CBCT and the position of the BAMP after surgery was investigated to evaluate the accuracy between virtual simulation and actual surgery. Twenty points were randomly selected from BAMP and used as reference points to investigate the single linear deviation, reported in mm, along the *x*-, *y*-, and *z*-axis. The *x*-axis is the transverse (lateral/medial) direction, the *y*-axis is the sagittal (anterior/posterior) direction, and the *z*-axis is the axial (cranial/caudal) direction. The deviation in the cranial, anterior, and lateral directions of the BAMP was considered positive (+), and the deviation in the contralateral direction was considered negative (−). The 3D average distance is expressed in mm, and the difference is indicated by color (Supplementary Figure S1). The 3D geometrical deviation was assessed after color coding high or low geometrical deviation using Geomagic software (Freeform Plus, 3D Systems, Morrisville, NC, USA) (Supplementary Figure S2).

## 3. Results

Four patients were screened, and two were included in this study for BAMP therapy. The two included patients were both male, 9 and 10 years old. All operations were performed under local anesthesia, and the average time from incision to the upper bone of the BAMP device through the transfer jig, from screw fixation to sutures, was 8.3 min per site, on average. Unlike for conventional plate surgery, the time to consider tooth germ injury and sufficiently dense bone and application work through screw bending were not relevant; therefore, the surgery time was dramatically reduced. In addition, since the plate fixing position was accurately known in advance, minimally invasive surgery, including incision and dissection, was possible.

During the follow-up, the customized miniplates showed no loosening or inflammation. The miniplates were well-retained for over a year, had sufficient retention force, and maintained good oral hygiene in the surrounding tissue.

Skeletal measurements revealed that BAMP decreased ANB difference (−4.56 to −1.09) and Wits appraisal (−7.52 to −3.26). Other measurements indices for sagittal and vertical growth were also in the normal range, indicating successful growth modification with customized BAMP therapy for skeletal Class III malocclusion (Table 1).

The accuracy between simulation and actual surgery was satisfactory. In the measurement of the linear deviation between preoperative simulation and actual surgery, the average difference was −0.0154 mm on the *x*-axis, −0.0946 mm on the *y*-axis, and 0.0579 mm on the *z*-axis, with an average distance of 0.1081 mm (Table 2).

## 4. Case Presentation

**Figure 1:**
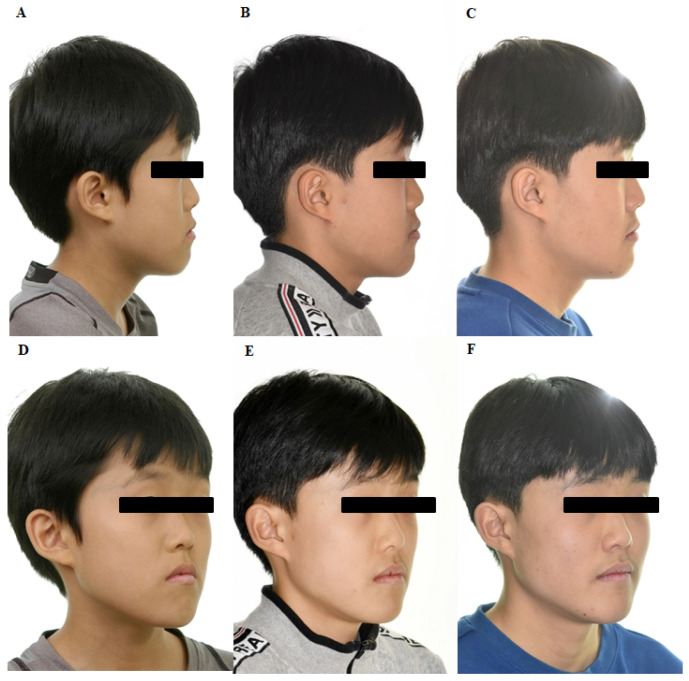
Facial photographs showing lateral and 45 degree profiles (**A**,**D**) at initial, (**B**,**E**) 12 months after customized bone-anchored maxillary protraction (BAMP) therapy, and (**C**,**F**) 24 months after customized BAMP therapy.

**Figure 2:**
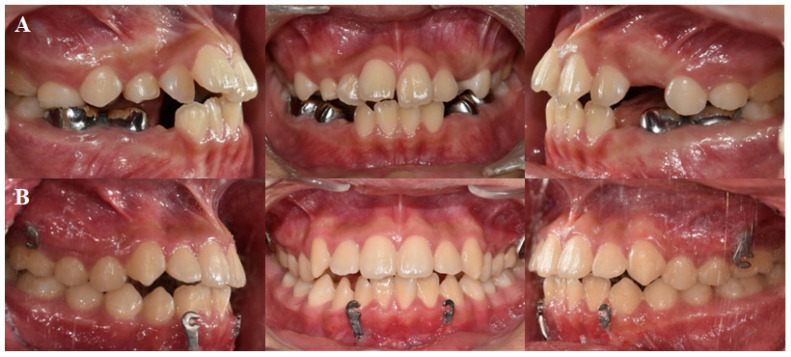
(**A**) Initial intraoral photographs. (**B**) Intraoral photographs 24 months after customized bone anchored maxillary protraction therapy showing Class I molar key.

A 10-year-old boy presented with maxillary retrognathism, mandible prognathism, and dentofacial deformity and was diagnosed with skeletal Class III malocclusion (Figure 1 and Figure 2). The patient had no systemic diseases and no signs of temporomandibular joint dysfunction. Cephalometric analysis showed a concave facial profile and hyperdivergent growth pattern. Considering the patient’s age and growth patterns, treatment with customized BAMP miniplates was considered as the most optimal therapy.

**Figure 3:**
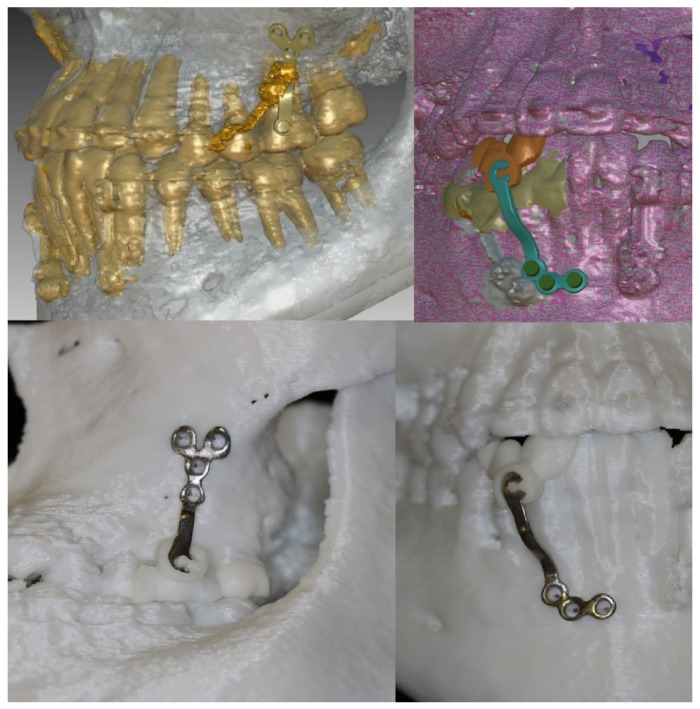
Preoperative simulation and application of fabricated customized plates and transfer jig on the individualized rapid prototyping model.

**Figure 4:**
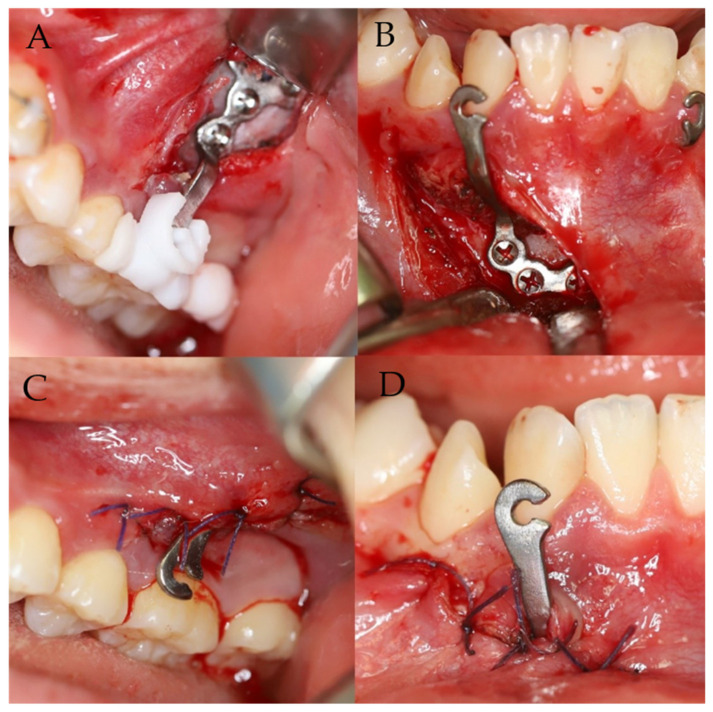
Surgical photographs. (**A**,**B**) Customized BAMP miniplates were transferred to the pre-simulated location using a transfer jig. (**C**,**D**) Postoperative photographs.

For the left maxilla, a ‘Y’ shaped plate was used and fixed at the zygomatic process to avoid possible disruption of the molar roots. An ‘L’ shaped plate was designed for the mandible and fixed at the symphysis of mandible. After preoperative simulation and fabrication, BAMP plates were used for the individualized rapid prototyping model (Figure 3). Under local anesthesia, customized BAMP plates were installed at the simulated location using the transfer jig as a guide. The jig was precisely matched with the reference point for accurate installation of the miniplates (Figure 4). The mean surgical time from incision to suture was 6.4 min for each side.

**Figure 5:**
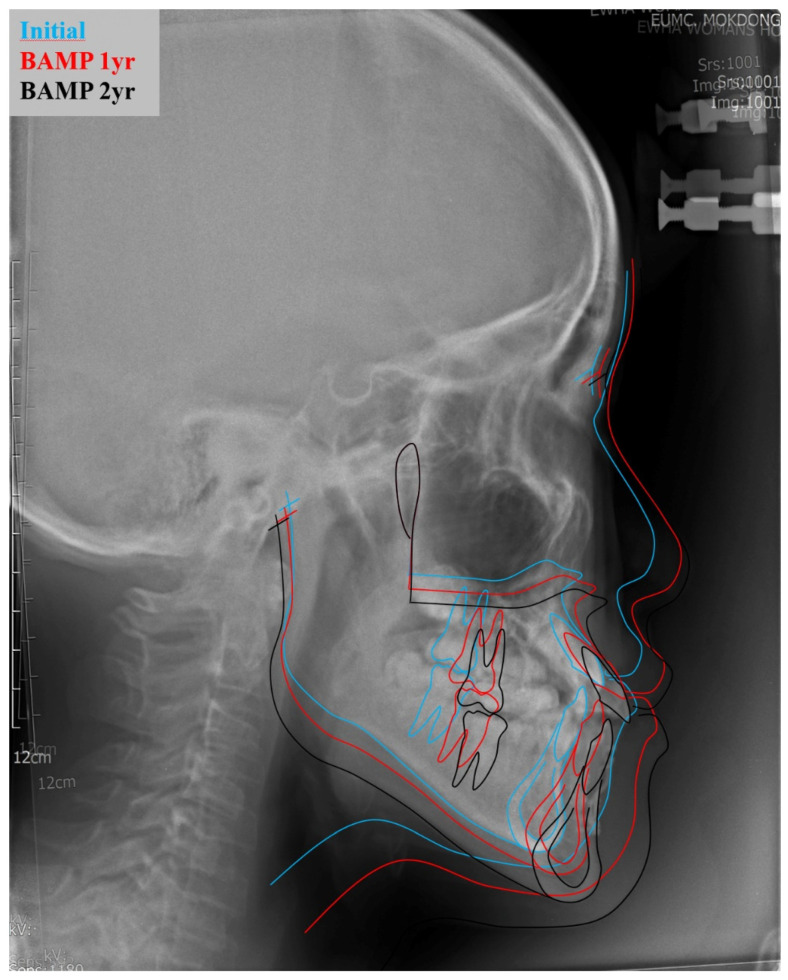
Superimposition of lateral cephalograms at initial stage, 12 months after BAMP therapy, and 24 months after BAMP therapy. The superimposition was based on the cranial base.

The patient was advised to maintain good oral hygiene after the operation. After soft tissue healing, Class III elastics (5/8 3.50 oz) were applied with an initial force of ~70 g on each side. The force of elastics (left: 5/16 3.50 oz; right: 3/16 3.50 oz) was increased to 150 g after 1 month of traction and again increased to 250 g after 3 months. The patient was instructed to wear the elastics full-time, except during meals and brushing. The patient also had to replace the elastics daily, with regular follow-up (Figure 4). During the follow-up, the customized miniplates showed no loosening or inflammation. The miniplates were well-retained for over two years, showing sufficient retention force, and maintained good oral hygiene in the surrounding tissue. Superimposition of lateral cephalograms indicated that BAMP therapy successfully protracted the maxilla and prevented mandibular growth and hyperdivergent vertical growth (Figure 5).

## 5. Discussion

Recently, bone-anchored maxillary protraction has been regarded as an effective treatment method for growth modification in growing skeletal Class III patients; however, an individualized approach, especially using 3D metal plate printing, has not been previously reported. The authors believe sharing our experience in the study will contribute to the acceleration of the medical use of individualized metal printing to the field of dentistry.

Skeletal Class III malocclusion correction in growing patients is considered one of the most challenging treatments in orthodontics. It is conducted using either an extraoral or intraoral device. The use of a facemask has been one of the most common approaches for maxillary traction, but it may result in undesired dentoalveolar outcomes, such as upper incisors or extrusion of the upper molars. A chin cup is effective for mandibular development restriction, but it may cause anterior teeth inclination in the mandible due to the pressure applied on the bone and soft tissue [13]. Wearing an extraoral appliance may be esthetically unpleasing and may cause social discomfort in growing patients, oftentimes leading to noncompliance [14]. Miniscrews or miniplates have replaced the use of extraoral devices for such orthopedic force transfer. Intraoral miniscrews and miniplates have shown sufficient biocompatibility and durability for the loading of orthodontic force, significantly influencing the advancement of maxillary structures [9,11].

However, the use of bone anchorages has been increasing, and various factors relating to the failure of implants are being studied. The location of the implant, diameter of the screw, shape of the miniplate, inflammation, and keratinization of peri-implant tissues affect the stability of bone anchorage [15,16]. Age is also a crucial factor in adolescent patients. The stability of BAMP therapy can be attributed to mechanical retention and the density and thickness of the cortical bone. In our case, the plate on the left maxilla and right mandible were found mobile after 5 months of installation and eventually failed. As a child, the patient’s developing bone may have not been strong enough to endure the force applied for anchorage. Applied orthopedic force for bone correction, which is relatively higher than that applied for tooth movement, may also have influenced the stability of bone anchorage. Bone remodeling occurs quickly and plays a significant role in young patients during their growing period. When excess force is applied on the anchorage device, its loading can significantly influence the density and turnover of the alveolar bone, loosening the BAMP correction device and leading to eventual failure.

Oral hygiene and plaque accumulation may also affect the success of plate insertion. Microbial flora at the interface between the artificial material and mucosal epithelium influence plate fixation. Anaerobic bacteria at the insertion site may cause inflammation. In our case, only mild infection was observed in the right mandible. Keeping the emergence point within the attached gingiva is essential to avoid bacterial invasion [17].

A customized titanium miniplate was manufactured after the failure of anchorage on the left maxilla and right mandible. The transfer jig created for each surgical site allowed clinicians to precisely determine the final location and delivery of the plate during the surgery [18,19]. CBCT and oral scan data were used to evaluate and design the customized miniplates accordingly. In the second operation, the customized miniplates were to be fixed at the zygomatic process and symphyseal to avoid the previous insertion site and any possible damage to the surrounding structures, such as the root and tooth germ. Bending and adjusting the plate contour not only alters the mechanical strength of the plate but also causes its inevitable deformation. Customized plates manufactured according to the skeletal contour of the patient eliminate the need for adjustment during the surgery. The use of customized devices allows clinicians to make smaller but more accurate incisions and deliver the plates in their ideal locations within a shorter period. There was a report of a minimally invasive approach for BAMP placement without surface incisions in the gingival margins or papillae. This potentially minimizes the gingival recession that sometimes accompanies flap surgery [20]. A combination of maxillary protraction, expansion and contraction would be beneficial in this case.

A BAMP device ideally fixes to the jawbone and directly contacts the oral surface through the open mucosa. There are inherent risks of plaque accumulation and chronic inflammation, but BAMP therapy is shorter and more efficient than extraoral orthopedic devices such as a chin cup.

## 6. Conclusions

Preoperative simulation and a 3D titanium printing technique allowed the fabrication of customized BAMP devices and their precise application at the planned locations. The clinical results demonstrated the stability and effectiveness of this modality for the treatment of skeletal Class III malocclusion.

## Figures and Tables

**Table 1:** Skeletal measurements: preoperative and postoperative (24 months) *.

Measurement	Reference Value	Preoperative	Postoperative 24 Months
Maxillo-mandibular measurement			
SNA	81.6 (3.1)	77.98	84.63
SNB	79.1 (3.0)	82.54	85.72
ANB difference	2.4 (1.8)	−4.56	−1.09
Wits appraisal	−2.7 (2.4)	−7.52	−3.26
Angular measurement			
FMA	29.63 (5.66)	30.34	28.98
SN-GoMe	36 (4)	37.1	32.72
A point—N Perpend	0.4 (2.3)	−5.37	−1.86
Facial height measurement			
Facial Height Ratio	65 (9)	61.7	66.37
Posterior Facial Height	85 (5.5)	76.84	90.76
Anterior Facial Height	127.4 (5.6)	124.52	136.74
Dentoalveolar measurement			
U1 to SN	107 (6)	123.45	120.62
IMPA	95.9 (6.3)	78.2	78.3
Interincisal angle	124 (8.3)	121.2	128.32
Soft tissue measurement			
Upper Lip E-plane	0.1 (2)	0.44	−1.58
Lower Lip E-plane	0.1 (2)	2.69	0.26
Nasolabial angle	84.9 (5)	93.86	101.9

* Values are presented as mean (standard deviation).

**Table 2:** Linear deviations in the *x*-, *y*-, and *z*-axis at 20 selected reference points.

Linear Deviation				
	*x*-Axis (Lateral/Medial)	*y*-Axis (Anterior/Posterior)	*z*-Axis (Cranial/Caudal)	Distance Difference
N	20	20	20	20
Median	−0.0154	−0.0946	0.0579	0.1081
Range	−0.1729–0.2448	−1.1643–1.5076	−0.9189–0.7126	0.0211–1.3772
SD	0.1463	0.9012	0.5515	0.5074

## Data Availability

Data is available with permission of corressponding author.

## Supplementary Materials

The following are available online at https://www.mdpi.com/article/10.3390/jpm11111087/s1, Figure S1: Distance measurement of 20 selected reference points, Figure S2: A color-coded deviation figure that visualizes the high or low geometrical deviation.
