# Supplementary material for: Individualized 3D-Printed Bone-Anchored Maxillary Protraction Device for Growth Modification in Skeletal Class III Malocclusion
Source: J Pers Med. 2021 Oct 26;11(11):1087. doi: 10.3390/jpm11111087 (PMC8619151; doi:10.3390/jpm11111087)
Supplement: Supplementary file 1 [file jpm-11-01087-s001.zip › jpm-1417043-supplementary.pdf]

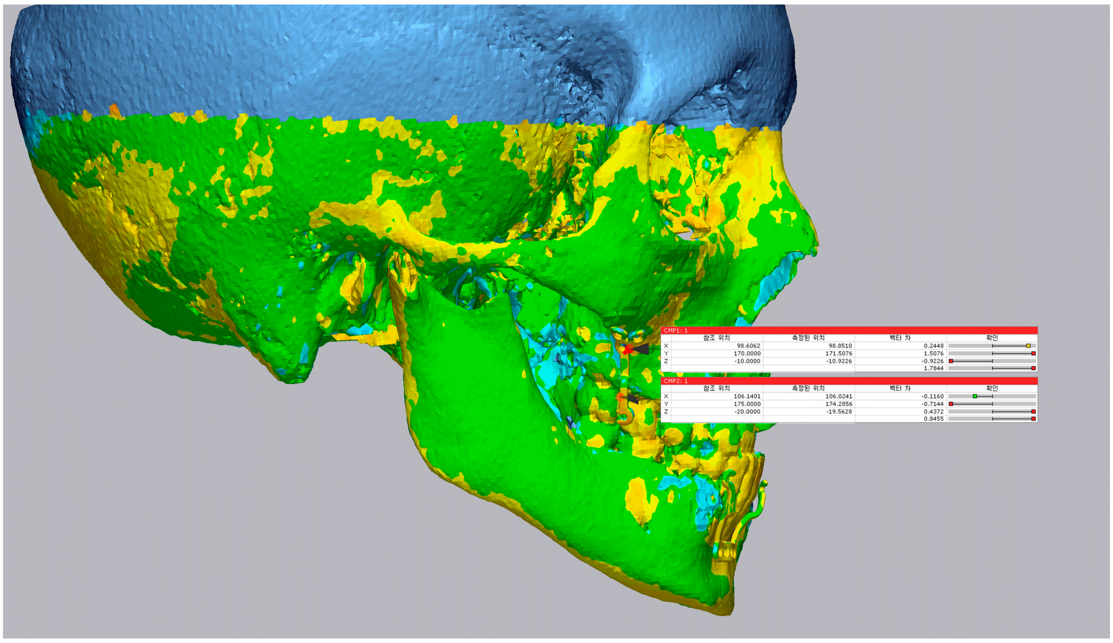

Figure S1. Distance measurement of 20 selected reference points.

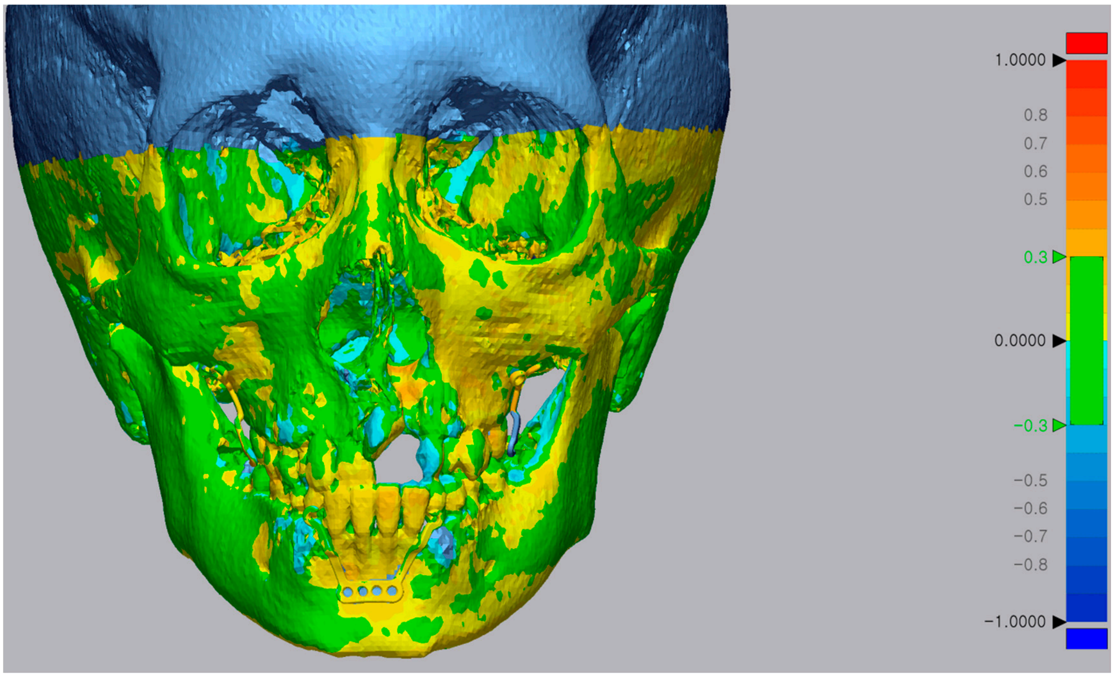

Figure S2. A color-coded deviation figure that visualizes the high or low geometrical deviation.
